# Verbascoside Protects Gingival Cells against High Glucose-Induced Oxidative Stress via PKC/HMGB1/RAGE/NFκB Pathway

**DOI:** 10.3390/antiox10091445

**Published:** 2021-09-12

**Authors:** Pei-Fang Hsieh, Cheng-Chia Yu, Pei-Ming Chu, Pei-Ling Hsieh

**Affiliations:** 1 Department of Urology, E-Da Hospital, Kaohsiung 82445, Taiwan; n52022@gmail.com; 2 Department of Medical Laboratory Science and Biotechnology, Chung-Hwa University of Medical Technology, Tainan 71703, Taiwan; 3 Institute of Oral Sciences, Chung Shan Medical University, Taichung 40201, Taiwan; ccyu@csmu.edu.tw; 4 Department of Dentistry, Chung Shan Medical University Hospital, Taichung 40201, Taiwan; 5 School of Dentistry, Chung Shan Medical University, Taichung 40201, Taiwan; 6 Department of Anatomy, School of Medicine, China Medical University, Taichung 404333, Taiwan; pmchu@mail.cmu.edu.tw

**Keywords:** verbascoside, PKC, HMGB1, impaired wound healing, antioxidant activity

## Abstract

Impaired wound healing often occurs in patients with diabetes and causes great inconvenience to them. Aside from the presence of prolonged inflammation, the accumulation of oxidative stress is also implicated in the delayed wound healing. In the present study, we tested the effect of verbascoside, a caffeoyl phenylethanoid glycoside, on the improvement of cell viability and wound healing capacity of gingival epithelial cells under high glucose condition. We showed that verbascoside attenuated the high glucose-induced cytotoxicity and impaired healing, which may be associated with the downregulation of oxidative stress. Our results demonstrated that verbascoside increased the activity of the antioxidant enzyme SOD and reduced the oxidative stress indicator, 8-OHdG, as well as apoptosis. Moreover, verbascoside upregulated the PGC1-α and NRF1 expression and promoted mitochondrial biogenesis, which was mediated by suppression of PKC/HMGB1/RAGE/NFκB signaling. Likewise, we showed the inhibitory effect of verbascoside on oxidative stress was via repression of PKC/HMGB1/RAGE/NFκB activation. Also, our data suggested that the PKC-mediated oxidative stress may lead to the elevated production of inflammatory cytokines, IL-6 and IL-1β. Collectively, we demonstrated that verbascoside may be beneficial to ameliorate impaired oral wound healing for diabetic patients.

## 1. Introduction

Diabetes mellitus is a multifaceted metabolic disorder that represents a major concern globally. According to the International Diabetes Federation (IDF), it was estimated that there were approximately 415 million people with diabetes worldwide, and by 2040, the predicted number may rise to 642 million [1]. A significant amount of health expenditure due to diabetes becomes a disease burden to be reckoned with. Furthermore, diabetes and its complications, such as macrovascular and microvascular diseases, constitute major causes of morbidity and mortality [2]. This chronic inflammatory disease not only affects the cardiovascular system, but it also causes dysregulation of wound healing, including in the oral tissues [3]. Diabetes has been known to be a risk factor of periodontitis, which is characterized by the destruction of the supporting structures of the teeth [4]. It has been indicated that diminished cell proliferation and migration, the elevation of pro-inflammatory cytokines, and reduced formation of new connective tissue and bone were observed in diabetic oral wound healing [3]. Nevertheless, the molecular mechanisms underlying this impaired healing process remain poorly understood.

During gingival healing, epithelial cells migrate to the injured site to cover denuded surfaces, and basal epithelial cells adjacent to migrating cells start to proliferate for wound closure [5,6]. Defective gingival healing of diabetic patients may cause invasion of microbes or other agents into tissues, which leads to chronic infection and aggravate the inflammatory condition. Apart from inflammation, mounting evidence has shown that oxidative stress is another contributing factor for prolonged diabetic wound healing [7]. As a key mediator of oxidative stress and inflammation, the role of the high mobility group box-1 (HMGB-1), a representative damage-associated molecular pattern (DAMP), in diabetes is gaining increasing attention [8]. It has been shown that hyperglycemia-induced reactive oxygen species (ROS) elevates the expression of HMGB1 and the receptor for advanced glycation end products (RAGE) in human aortic endothelial cells [9]. In addition, HMGB-1 signaling via RAGE has been thought to promote the production of pro-inflammatory cytokines through the activation of nuclear factor-κB (NF-κB) [10]. Whether this HMGB1/RAGE/NF-κB cascade involves in the disturbed oral wound healing in diabetes remains to be determined.

Verbascoside, also known as acteoside (C_29_H_36_O_15_), is a phenylethanoid glycoside isolated from various herbal plants, such as *Verbascum phlomoides* [11] or *Buddleja globose* [12]. It has been known to possess numerous bioactivities, such as anti-tumor, anti-viral, anti-inflammatory, and anti-oxidant properties [13]. Besides, verbascoside has been shown to serve as a protein kinase C (PKC) inhibitor [14]. It has been revealed that elevation of PKC in fibroblasts derived from diabetic patients contributed to impaired skin wound healing [15]. Since HMGB1 can be phosphorylated by PKC for secretion [16], we aimed to investigate the effect of verbascosides on the expression of PKC/HMGB1/RAGE/NF-κB signaling and if verbascosides treatment improved the compromised wound repair of gingival cells under high glucose condition through suppression of oxidative stress and inflammation.

## 2. Materials and Methods

### 2.1. Cells and Chemicals

The Smulow-Glickman (S-G) gingival epithelial cell line used in this study was originally derived from human attached gingiva [17], and obtained from Kasten et al., East Tennessee State University, Quillen College of Medicine, Johnson City, TN, USA [18]. Cells were maintained in Dulbecco’s modified Eagle’s medium (DMEM; 11965092, Thermo Fisher Scientific, Waltham, MA, USA) supplemented with 10% fetal bovine serum (Gibco, Grand Island, NY, USA) at 37 °C in the presence of 5% CO_2_ and medium were changed every 2–3 days. For high glucose groups, cells were exposed to 70 mmol/L concentration of D-glucose for 24 h followed by 25, 50, 100 μM verbascoside (00820580, HWI Analytik Gmbh, Rulzheim, Germany) or 5 mM N-Acetyl-L-cysteine (NAC; A9165, Sigma–Aldrich, St. Louis, MO, USA) for another 24 h. Bisindolylmaleimide I (Bis I; 5 μM) was purchased from Calbiochem (La Jolla, CA, USA) and used as a PKC inhibitor [19]. Glycyrrhizic acid (200 μM), FPS-ZM1 (1 μM), and dehydroxymethylepoxyquinomicin (DHMEQ) (10 μg/mL) were all obtained from Sigma-Aldrich and used as a HMGB1 inhibitor [20], a RAGE antagonist [21], and a NF-κB inhibitor [22], respectively. The concentrations of Bis I, glycyrrhizic acid, FPS-ZM1 and DHMEQ were referred from published studies [20,23,24,25].

### 2.2. Prestoblue, MTT, and LDH Assays

PrestoBlue™ Cell Viability Reagent (A13262, Invitrogen, Carlsbad, CA, USA) was utilized for cell viability and this assay was carried out in a 96-well plate according to the manufacturer’s instructions. Briefly, each well contained the cells to be tested with cultured medium or verbascoside-containing medium and was incubated at 37 °C for 2 h. After incubation of 100 μL 10X PrestoBlue reagent, the absorbance was measured at 570/600 nm using iMark™ Microplate Absorbance Reader (Bio-Rad, Hercules, CA, USA).

MTT assay was conducted to measure cell proliferation. Cells were seeded into a 24-well plate for 24 h and then treated with various concentrations of verbascoside for another 24 h. Afterward, the cells were incubated with 3-(4,5-dimethylthiazol-2-y1)-2,5-diphenyltetrazolium bromide (MTT) in a humidified atmosphere containing 5% of CO_2_ at 37 °C. The blue formazan crystals of viable cells were dissolved using isopropanol and then evaluated spectrophotometrically at 595 nm.

Lactate dehydrogenase (LDH) Cytotoxicity Detection Kit (630117, Clontech Laboratories, Mountain View, CA, USA) was used to assess the level of LDH released from the plasma membrane of damaged cells according to the manufacturer’s instructions. Briefly, cells were added to a 96-well plate and cultured at 37 °C in 5% CO_2_ overnight. The supernatant was then collected and transferred to another clear 96-well plate, and 100 μL of the reaction mixture from the LDH assay kit was added to each well for 30 min. Cells treated with 1% Triton X-100 were used as a positive control group for maximum LDH release. Absorbance was then measured at 490 nm (reference wavelength greater than 600 nm).

### 2.3. Wound Healing Assay

Cell suspension was added into two chambers of the silicone Culture-Insert 2 Well in µ-Dish 35 mm (ibidi GmbH, Martinsried, Germany), and cells were allowed to adhere and spread on the substrate (cells in two chambers were separated by 500 µm). After removing the culture-insert, cultured medium or verbascoside-containing medium were provided. Each well was photographed at 0 and 48 h to capture the two different fields of each group at each time point.

### 2.4. Examination of Antioxidant Capacity and Oxidative Stress

Superoxide Dismutase (SOD) Activity Colorimetric Assay Kit (K335-100, BioVision, Milpitas, CA, USA) was used for the examination of antioxidant capacity by assessing the water-soluble formazan dye upon reduction with superoxide anion to detect the inhibition activity of SOD. Briefly, the WST-1 working solution and the SOD enzyme working solution were added to the wells and mixed thoroughly. After incubation for 20 min, the inhibition activity of SOD was estimated using a microplate reader at 450 nm. The SOD activity (inhibition rate %) was calculated according to the manufacturer’s instructions.

8-hydroxy 2 deoxyguanosine (8-OHdG) ELISA Kit (ab201734, Abcam, Cambridge, UK) was utilized to measure the oxidative damage of DNA by oxidative stress according to the manufacturer’s protocol. Briefly, standards and samples were added into each well as instructed followed by incubation of HRP conjugated 8-OHdG antibody preparation for an hour. The enzymatic color reaction was developed after the TMB substrate solution was added and then the absorbance was measured at 450 nm.

### 2.5. Western Blot

Cells were lysed by lysis buffer (10 mM Tris, 1 m MEDTA,1% Triton X-100, 1 mM Na_3_VO_4_, 20 μg/mL aprotinin, 20 μg/mL leupeptin, 1 mM dithiothreitol, and 50 μg/mL phenylmethylsulfonyl fluoride). Cell lysates were subjected to 10% SDS-PAGE and proteins were transferred to the PVDF membrane by an electro-transfer unit. Membranes were probed with primary antibodies overnight at 4  °C. The primary antibodies included anti-Bcl-2 (ab692, Abcam; 1:2000), anti-caspase-3 (ab32351; 1:2000), anti-Bax (#89477, cell signaling technology, Beverly, MA, USA; 1:2000), anti-PGC1-α (ab54481; 1:2000), anti-NRF1 (ab34682; 1:2000), anti-PKC (ab181558; 1:2000), anti-HMGB1 (ab18256; 1:2000), anti-RAGE (ab216329; 1:2000), and anti-NFκB (ab16502; 1:2000). Following incubation of primary antibodies, the membranes were rinsed three times and incubated with corresponding secondary antibodies. The immunoreactive bands were developed using a Western-Ready™ ECL Substrate Plus Kit (426316, BioLegend, San Diego, CA, USA) and detected by MultiGel-21^®^ image system. β-actin was used as an internal control.

### 2.6. Assessment of Mitochondrial Biogenesis

Mitochondrial biogenesis was analyzed using MitoBiogenesis™ InCell ELISA Kit (ab110217) according to the manufacturer’s protocol. Briefly, cells were seeded in a 96-well plate and fixed with 4% paraformaldehyde after adhering to the bottom of the plate. 0.5% acetic acid was added for 5 min to block endogenous alkaline phosphatase activity. Afterward, 0.1% Triton X-100 was used to permeabilize cells followed by 2X blocking solution addition. These cells were then incubated with primary antibodies against mtDNA encoded COX-I, and nuclear-DNA encoded SDHA proteins. After washing, the reaction was sequentially developed first with AP reagent and then HRP development solution. A 15-min kinetic reaction with a 1 min interval was recorded using a microplate reader at 405 nm (for AP detection of SDH-A) and 600 nm (for HRP detection of COX-I). The ratio of COX-I/SDHA was calculated to determine mitochondrial biogenesis.

### 2.7. Quantitative Real-Time PCR (qRT-PCR)

Total RNA of each sample was extracted using an Absolutely PureLink™ RNA Mini Kit (Invitrogen Life Technologies, Carlsbad, CA, USA) and subjected to RT by SuperScript™ First-Strand Synthesis System (Invitrogen) for RT-PCR. All cDNA samples were diluted as a working template in TaqMan™ Fast Advanced Master Mix (Applied Biosystems, Waltham, MA, USA), which was carried out on an ABI 7500 Fast Real-Time PCR System (Applied Biosystems). PCR amplification was conducted by using the TaqMan primers and probes (Assay ID: Hs00176973_m1 for PKC α; Assay ID: Hs01923466 for HMGB1; Assay ID: Hs00179504 for RAGE; Assay ID: Hs00765730 for NF-κB) using the TaqMan Universal PCR Master Mix Protocol (Applied Biosystems) according to the manufactures instructions.

### 2.8. Measurement of Inflammatory Cytokine Production

The secretion of IL-6 (BSKH1007) and IL-1β (BSKH1001) were determined using an enzyme-linked immunosorbent assay (ELISA) kit (Bioss, Woburn, MA, USA) and quantified at 450 nm according to the manufacturer’s instructions. Cells were cultured in 6-well plates with verbascoside, NAC, or PKC inhibitor for 24 h. Cell supernatants were collected and centrifuged to remove dead cells. Each sample was analyzed in triplicate.

### 2.9. Statistical Analysis

The results were expressed as mean ± SEM. Statistical analyses were performed using one-way ANOVA followed by an LSD post hoc test as appropriate. A *p*-value < 0.05 was considered statistically significant.

## 3. Results

### 3.1. Treatment of Verbascoside Mitigates the Suppressed Cell Proliferation and Wound Healing Capacity of Gingival Epithelial Cells under High Glucose Condition

**Figure 1:**
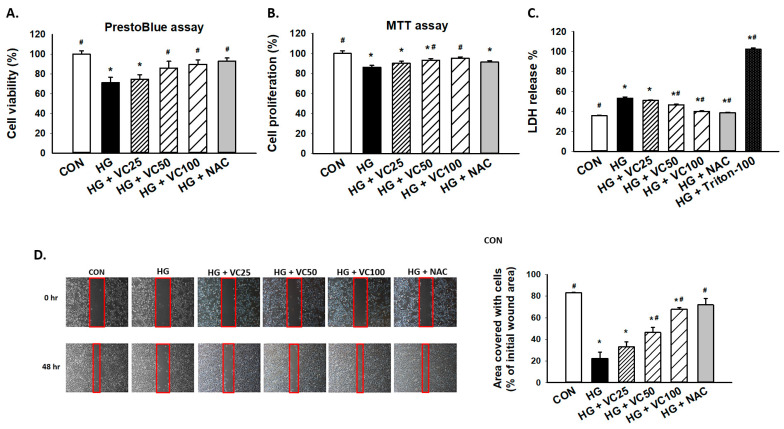
Verbascoside prevents the high glucose-inhibited cell proliferation and wound healing capacity of gingival epithelial cells. (**A**) PrestoBlue and (**B**) MTT assays were carried out to examine the cell viability of high glucose-treated cells in response to various concentrations of verbascoside (25, 50 and 100 μM) or N-acetyl-l-cysteine (NAC; 5 mM); (**C**) LDH assay was conducted to show that verbascoside protected cells against high glucose-induced cytotoxicity. Triton X-100 served as a positive control group; (**D**) wound healing capacity was tested using a two-well culture-insert. * indicates *p*  <  0.05 compared to the control group (CON); # indicates *p*  <  0.05 compared to the high glucose group (HG).

To assess the cytoprotective effect of verbascoside, PrestoBlue and MTT assays were employed to examine cell viability and proliferation of high glucose-treated S-G gingival epithelial cells. As shown in Figure 1A,B, both PrestoBlue and MTT assays showed that verbascoside dose-dependently reversed the repressed cell proliferation in high glucose-cultured cells. By using PrestoBlue assay, we observed that the cell death caused by high glucose was attenuated following the treatment of a commonly used antioxidant N-acetyl-l-cysteine (NAC), suggesting that oxidative stress may mediate the downregulation of cell viability in response to high glucose. Likewise, lactate dehydrogenase (LDH) cytotoxicity assay demonstrated that incubation of high glucose-treated gingival epithelial cells with verbascoside markedly suppressed LDH release and improved cell survival in a dose-dependent manner, and NAC exhibited a similar effect (Figure 1C). Besides, epithelial cells migrate and proliferate at the injured site during the healing process, and we showed that these capacities of epithelial cells were inhibited under the high glucose condition (Figure 1D). Nevertheless, these impairments were improved in high glucose-cultured groups by adding verbascoside or NAC (Figure 1D). These results indicated the beneficial effect of verbascoside against high glucose-induced cell death and disturbed wound healing ability.

### 3.2. Verbascoside Attenuates the Oxidative Stress and Apoptosis in High Glucose-Cultured Gingival Epithelial Cells

**Figure 2:**
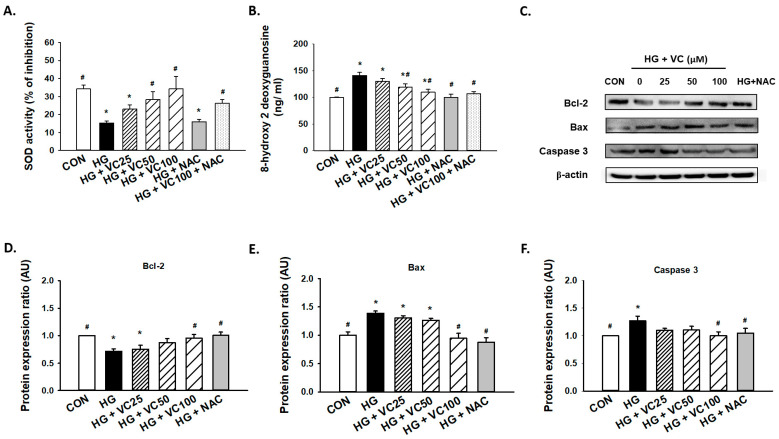
Verbascoside diminishes the high glucose-induced oxidative stress and apoptosis of gingival epithelial cells. (**A**) superoxide dismutase (SOD) and (**B**) 8-hydroxy-2-deoxyguanosine (8-OHdG) assays were utilized to assess the antioxidant capacity and oxidative stress of high glucose-cultured cells following treatment with various concentrations of verbascoside (25, 50 and 100 μM) or N-acetyl-l-cysteine (NAC; 5 mM); (**C**) representatives of western blots and densitometric analysis of Bcl-2 (**D**), Bax (**E**), and caspase 3 (**F**) in high glucose-treated cells incubated with various concentrations of verbascoside (25, 50 and 100 μM) or N-acetyl-l-cysteine (NAC; 5 mM) were presented. * indicates *p*  <  0.05 compared to the control group (CON); # indicates *p*  <  0.05 compared to the high glucose group (HG).

Superoxide dismutase (SOD) is an antioxidant enzyme that has been shown to be implicated in the protracted wound healing of diabetic rats [26]. We showed that verbascoside upregulated SOD activity of high glucose-cultured cells in a dose-dependent fashion, whereas administration of NAC alone failed to increase enzyme activity of SOD (Figure 2A), possibly due to the insufficient amount of NAC. Besides, 8-hydroxy-2-deoxyguanosine (8-OHdG), a byproduct of oxidative DNA damage, was found to be abundantly upregulated in high glucose-treated cells (Figure 2B). As expected, this indicator of oxidative stress in high glucose-cultured groups was markedly decreased in the presence of verbascoside and NAC (Figure 2B).

Furthermore, a concentration-dependent decrease in apoptosis was observed (Figure 2C). We demonstrated that administration of 100 μM verbascoside resulted in the upregulation of Bcl-2 (Figure 2C,D), and downregulation of Bax (Figure 2C,E) and caspase 3 (Figure 2C,F). Also, inhibition of oxidative stress by NAC displayed a comparable effect (Figure 2C–F). Overall, these findings supported that verbascoside may serve as a promising agent to alleviate high glucose-stimulated apoptosis in gingiva via repression of oxidative stress.

### 3.3. The High Glucose-Induced Mitochondrial Dysfunction Is Ameliorated by Verbascoside

**Figure 3:**
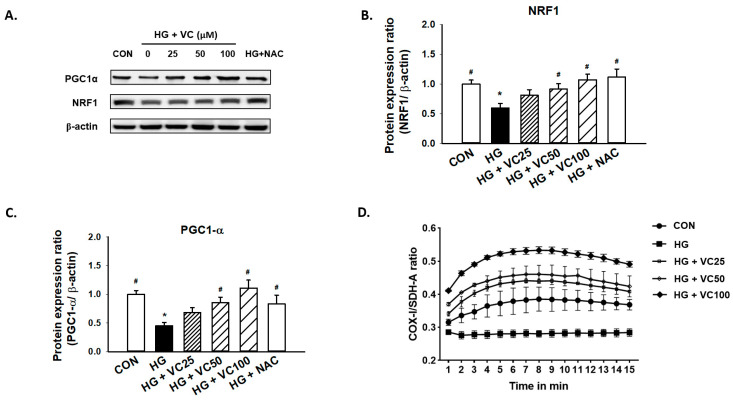
Verbascoside attenuates the high glucose-induced mitochondrial dysfunction of gingival epithelial cells. Representatives of Western blots (**A**), and densitometric analysis of PGC1-α (**B**) and NRF1 (**C**) in high glucose-treated cells incubated with various concentrations of verbascoside (25, 50 and 100 μM) or NAC (5 mM) were presented. (**D**) The ratio of COX1/SDH-A was used to show mitochondrial biogenesis over the course of 15 min. * indicates *p*  <  0.05 compared to the control group (CON); # indicates *p*  <  0.05 compared to the high glucose group (HG).

Peroxisomal proliferator activator receptor γ coactivator (PGC1)-α and nuclear respiratory factor 1 (NRF1) are primary regulators of mitochondrial biogenesis [27]. Mitochondrial dysfunction has been known to be associated with the increased reactive oxygen species (ROS) production [28], and it has been previously reported that both PGC1-α and NRF1 are reduced in diabetic subjects [29]. Here, we showed a consistent result that high glucose decreased the expression of PGC1-α and NRF1, while treatment of verbascoside dose-dependently increased the expression levels of these two factors (Figure 3A–C). Similarly, administration of NAC reversed the downregulation of NRF1 and PGC1-α (Figure 3A–C), indicating that the high glucose-inhibited PGC1-α and NRF1 expression may be due to the accumulation of oxidative stress.

Additionally, we assessed the ratio of cytochrome c oxidase I (COX1)/succinate dehydrogenase complex, subunit A (SDH-A) to measure mitochondrial biogenesis. COX-I is encoded by mitochondrial DNA (mtDNA) and its proper synthesis is greatly relied on mtDNA integrity [30]. SDH-A is encoded by nuclear DNA and its activity is typically not affected by impaired mtDNA [31]. As shown in Figure 3D, exposure to high glucose conditions caused suppression of mitochondrial biogenesis. However, treatment of various concentrations of verbascoside increased the COX1/SDH-A ratio (Figure 3D), suggesting that it may promote mitochondrial function, at least partially, through upregulation of PGC1-α and NRF1.

### 3.4. Verbascoside Inhibits the High Glucose-Elicited PKC/HMGB1/RAGE/NFκB Pathway

**Figure 4:**
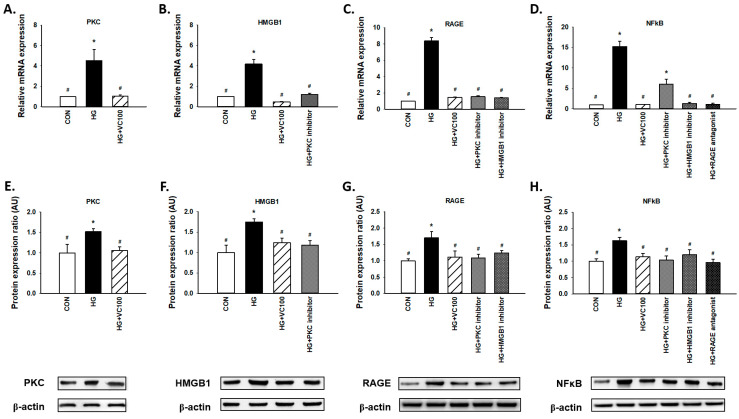
Verbascoside suppresses the high glucose-activated PKC/HMGB1/RAGE/NFκB pathway in gingival epithelial cells. Relative mRNA (**A**–**D**) and protein expression with representatives of Western blots (**E**–**H**) of PKC, HMGB1, RAGE, and NFκB in high glucose-stimulated cells with verbascoside treatment were presented. In some groups, coincubation with PKC inhibitor (Bis I; 5 μM), HMGB1 inhibitor (Glycyrrhizic acid; 200 μM), or RAGE antagonist (FPS-ZM1; 1 μM) was performed as suggested. * indicates *p*  <  0.05 compared to the control group (CON); # indicates *p*  <  0.05 compared to the high glucose group (HG).

Results from qRT-PCR (Figure 4A–D) and western blot (Figure 4E–H) revealed that both gene and protein expression levels of PKC, HMGB1, RAGE, and NFκB were suppressed by verbascoside. First, we observed that PKC was decreased in the verbascoside-treated cells (Figure 4A,E). Moreover, we showed the expression of HMGB1 was reduced by PKC inhibitor (Figure 4B,F), and the expression of RAGE was downregulated by PKC inhibitor and HMGB1 inhibitor (Figure 4C,G). Also, NFκB was suppressed by inhibitors of PKC and HMGB1 as well as the antagonist of RAGE (Figure 4D,H). Taken together, these results revealed that verbascoside possesses the inhibitory effect on the high glucose-induced PKC/HMGB1/RAGE/NFκB activation.

### 3.5. Suppression of PKC/HMGB1/RAGE/NFκB Signaling Reverses the Downregulation of PGC1-α and NRF1

**Figure 5:**
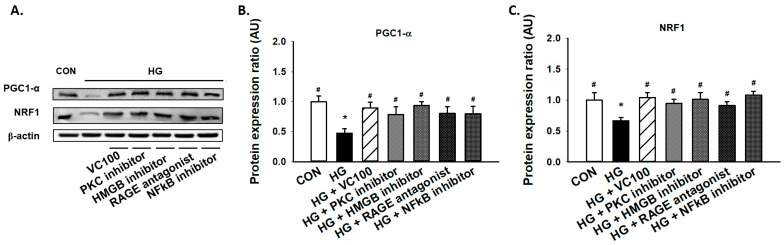
Downregulation of the PKC/HMGB1/RAGE/NFκB pathway promotes the high glucose-inhibited PGC1-α and NRF1 expression in gingival epithelial cells. Representatives of Western blots (**A**), and densitometric analysis of PGC1-α (**B**) and NRF1 (**C**) in high glucose-treated cells along with verbascoside, PKC inhibitor, HMGB1 inhibitor, RAGE antagonist, or NFκB inhibitor (DHMEQ; 10 μg/mL). * indicates *p*  <  0.05 compared to the control group (CON); # indicates *p*  <  0.05 compared to the high glucose group (HG).

Subsequently, we aimed to evaluate if the activation of the PKC/HMGB1/RAGE/NFκB pathway participated in the reduced expression of PGC1-α and NRF1 under high glucose conditions since it has been reported that PKC modulates mitochondrial function after oxidant injury in renal cells [32] and NF-κB regulates mitochondrial biogenesis by increasing the expression or activity of PGC-1α and NRF 1 [33]. We showed that inhibition of PKC reverted the reduction of PGC1-α and NRF1 caused by high glucose (Figure 5A–C). Similarly, we demonstrated that administration of HMGB1 inhibitor, RAGE antagonist, and NFκB inhibitor all upregulated the expression levels of PGC1-α and NRF1 in gingival epithelial cells in response to high glucose stimulation as the verbascoside-treated group (Figure 5A–C). These findings showed that blockade of the PKC/HMGB1/RAGE/NFκB pathway (e.g., administration of verbascoside) holds the potential to enhance mitochondrial function via upregulation of PGC1-α and NRF1.

### 3.6. Inhibition of the PKC/HMGB1/RAGE/NFκB Pathway Diminishes High Glucose-Induced Oxidative Stress and the Subsequent Inflammation

**Figure 6:**
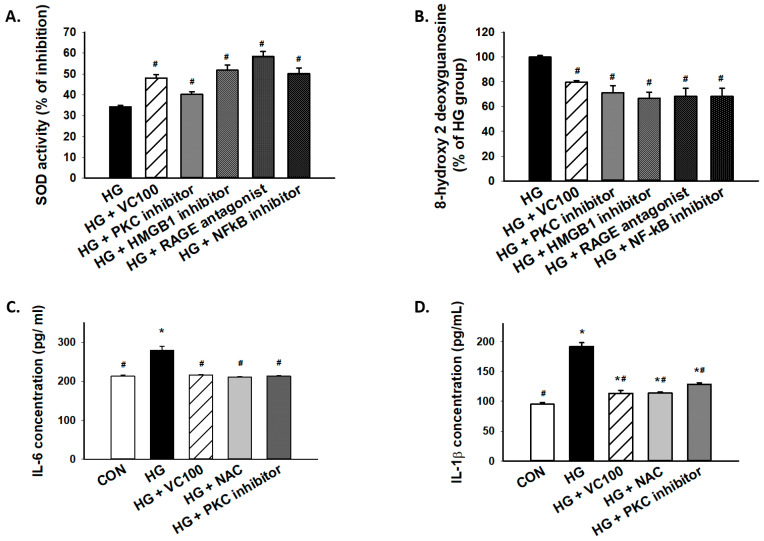
Inhibition of high glucose-induced oxidative stress and inflammation through suppression of PKC/HMGB1/RAGE/NFκB pathway by verbascoside. (**A**) SOD and (**B**) 8-OHdG assays were used to assess oxidative stress of high glucose-cultured cells following inhibition of PKC/HMGB1/RAGE/NFκB signaling; the high glucose-elicited production of IL-6 (**C**) and IL-1β (**D**) were downregulated in cells treated with verbascoside, NAC, or PKC inhibitor. * indicates *p*  <  0.05 compared to the control group (CON); # indicates *p*  <  0.05 compared to the high glucose group (HG).

Furthermore, we tested whether PKC/HMGB1/RAGE/NFκB signaling mediated the accumulation of oxidative stress in high glucose-cultured cells. Our results showed that the SOD activity was increased in verbascoside-treated cells or cells with inhibition of this signaling pathway (Figure 6A), whereas 8-OHdG production was markedly decreased in verbascoside-treated cells or cells with repression of PKC/HMGB1/RAGE/NFκB signaling (Figure 6B). Besides, we demonstrated that downregulation of oxidative stress (NAC-treated) successfully mitigated the high glucose-elicited production of interleukin (IL)-6 (Figure 6C) and IL-1β (Figure 6D), which was also observed in the PKC inhibitor-treated group (Figure 6C,D). As such, our results suggested that the high glucose-induced elevation of IL-6 or IL-1β was attributed, at least partially, to oxidative stress which was mediated by the PKC/HMGB1/RAGE/NFκB pathway.

## 4. Discussion

Verbascoside has been recognized as a widespread polyphenol with anti-oxidant and anti-inflammatory properties. For instance, it has been shown to attenuate the X-ray-induced intracellular ROS and apoptosis in human skin fibroblasts via mitogen-activated protein kinase (MAPK) signaling [34]. Another study demonstrated that verbascoside inhibited various inflammatory mediators in the primary rat chondrocytes treated with IL-1β through the MAPK pathway as well [35]. Moreover, recent studies have suggested verbascoside possessed the anti-diabetic potential since it has been reported to lower hyperglycemia and hyperlipidemia in diet/STZ-induced diabetic rats [36]. In a KK-Ay mouse model of type 2 diabetes, verbascoside was found to enhance lipolysis and fatty acid oxidation by inducing mRNA expression of adipose triglyceride lipase through the AMP-activated protein kinase (AMPK) pathway [37]. One of the more recent studies has suggested that verbascoside may be beneficial in terms of prevention and treatment of diabetes since it exerted protective effects against ER stress-associated dysfunctions in human β-cells via reduction of protein kinase RNA-like ER kinase (PERK) expression [38]. In the current study, we showed verbascoside modulated mitochondrial biogenesis and redox homeostasis via PKC/HMGB1/RAGE/NFκB pathway under high glucose conditions, leading to mitigation of inflammation and apoptosis.

It has long been known that high glucose increased diacylglycerol (DAG), which activated PKC [39]. Various studies have revealed that the DAG/PKC axis contributed to insulin resistance [40] and hyperglycemia-induced oxidative stress in diabetic rat kidneys [41]. Apart from DAG, it has been revealed that the accumulation of advanced glycation end products (AGEs) elevated the translocation of PKC and oxidative stress in rat neonatal mesangial cells as well [42]. Several lines of evidence suggested that PKC mediated the production of oxidative stress in diabetes via NADPH oxidase activation using aortic smooth muscle cells [43], retinal endothelial cells [44], and human renal mesangial cells [45]. In line with these findings, we also showed that high glucose-induced the expression of PKC in gingival epithelial cells and suppression of PKC resulted in an increase in anti-oxidant enzyme activity and a reduction of oxidative stress marker. On the other hand, it has been shown that high glucose induced NF-κB activity and inflammatory cytokine expression via toll-like receptor 2 (TLR2) and TLR4 expression in human monocytes [46]. Dasu et al. showed that the upregulation of TLR2 and TLR4 expression was through PKC-α and PKC-δ, respectively, by stimulating NADPH oxidase [46]. Another study demonstrated a similar finding showing that high glucose elevated the NF-κB expression and secretion of inflammatory cytokine through PKC-induced TLR2 pathway in gingival fibroblasts [47]. Here, we revealed that PKC can activate NF-κB by HMGB1/RAGE axis in addition to TLR2 pathway, which resulted in the subsequent aggravation of inflammation via generation of oxidative stress.

As a nuclear protein, HMGB1 has been found to trigger inflammation and oxidative stress [8,48]. It has been shown that HMGB1 can be phosphorylated by PKC and shuttled from nuclear to cytoplasmic compartments that direct it toward secretion [16,49]. The extracellular HMGB1 has been known to act as a multifunctional cytokine and induce NF-κB, leading to the secretion of various proinflammatory cytokines [8]. Emerging evidence has revealed the significance of the HMGB1/RAGE/NF-κB axis in diabetic complications. For instance, it has been shown that the retinal expression levels of HMGB1 and RAGE and the activity of NF-κB were elevated in the diabetic retina [50]. In addition, Sohn et al. demonstrated the direct binding of NF-κB p65 to the RAGE promoter using ChIP assays [50]. The HMGB1/RAGE/NF-κB signaling also contributed to neuroinflammation [51] and periodontitis [52] in diabetic mice. It has been revealed that high glucose prompted the translocation of HMGB1 from the nucleus to the cytosol through an NADPH oxidase and PKC-dependent pathway in vascular smooth muscle cells [53]. Also, they showed the upregulation of HMGB1 was crucial to high-glucose-induced vascular calcification through regulation of NFκB activation and bone morphogenetic protein-2 (BMP-2) [53]. Our results demonstrated that PKC/HMGB1/RAGE/NF-κB signaling may participate in diabetes-associated impairment of oral wound healing.

In the oral cavity, higher expression of HMGB1 was detected in gingival tissues and gingival crevicular fluid (GCF) of patients with periodontitis and peri-implantitis, which was accompanied by the elevated concentrations of pro-inflammatory cytokines, such as IL-1β, IL-6, and IL-8 [54]. In diabetic patients, the GCF level of IL-1β was increased and some reports suggested that the GCF IL-6 level was upregulated as well [55,56]. Besides, the increased mRNA expression of RAGE was found in the type 2 diabetes gingival epithelium [57]. It has been shown that gingival epithelial cells secreted HMGB1 upon stimulation of tumor necrosis factor-alpha (TNF-α) [58] or IL-1β [59]. Ito et al. reported that HMGB1 and RAGE were markedly expressed in gingiva and promptly released during gingival inflammation [59]. Another study showed that the HMGB1/RAGE axis involved in the intraoral palatal wound healing and knockdown of RAGE inhibited the cell migration and cell proliferation in gingival epithelial cells [60]. Our results were consistent with these findings and showed that the HMGB1/RAGE axis and pro-inflammatory cytokines were upregulated in high glucose-stimulated gingival epithelial cells.

It is becoming increasingly evident that the high glucose-induced inflammation and oxidative stress may contribute to the impaired healing processes. The elevation of pro-inflammatory cytokines, such as IL-1β, has been found to impair diabetic wound healing [61]. The high glucose-induced oxidative stress also has been shown to impede the proliferation and migration of human gingival fibroblasts [62]. In agreement with these results, we showed that suppression of the PKC/HMGB1/RAGE/NFκB-mediated oxidative stress and pro-inflammatory cytokine production may improve the high glucose-inhibited cell proliferation and wound healing. Furthermore, we demonstrated that this signaling pathway was also associated with high glucose-related mitochondrial dysfunction, which was pivotal to oxidative stress and apoptosis in various diabetic complications [63,64].

## 5. Conclusions

**Figure 7:**
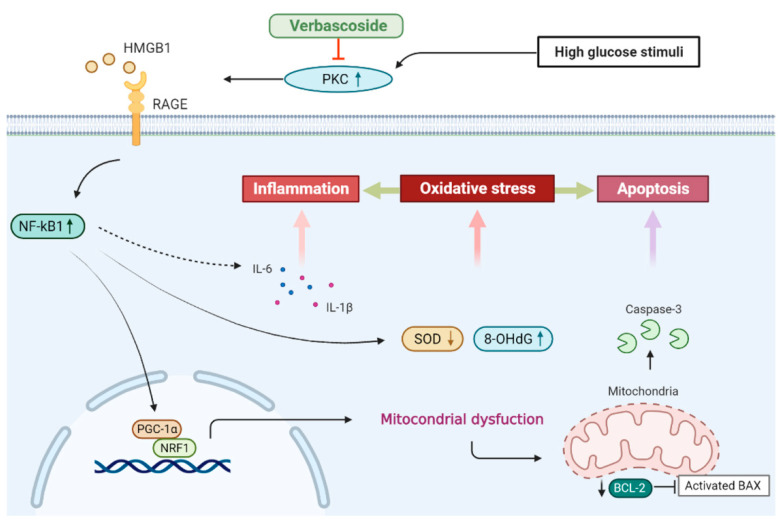
Schematic diagram showing the possible mechanism underlying the protective effect of verbascoside on high glucose-induced impairment of oral wound healing.

In this study, we showed that administration of verbascoside improved the cell viability and wound healing capacity of gingival epithelial cells in a dose-dependent fashion under high glucose conditions, which may be associated with the downregulation of oxidative stress. Our results demonstrated that verbascoside dose-dependently increased the anti-oxidant enzyme, SOD, and reduced the DNA damage marker, 8-OHdG, along with suppression of apoptosis in high glucose-cultured cells. We showed that the treatment of verbascoside reversed the high glucose-inhibited PGC1-α and NRF1 expression as well as promoted mitochondrial biogenesis. Furthermore, our data suggested that these effects were mediated by downregulation of the PKC/HMGB1/RAGE/NFκB pathway. Also, we demonstrated that a decrease of oxidative stress by verbascoside via PKC signaling mitigated the high glucose-induced inflammatory cytokines, both IL-6 and IL-1β (Figure 7). Taken together, this study revealed that administration of verbascoside may be beneficial to individuals with diabetes in terms of amelioration of impaired oral wound healing.

## Data Availability

The data is contained within this article.
